# Elevated total bile acid levels as an independent predictor of mortality in pediatric sepsis

**DOI:** 10.1038/s41390-024-03438-3

**Published:** 2024-09-12

**Authors:** Yanfei Wang, Kelei Deng, Peiquan Lin, Limin Huang, Lei Hu, Jing Ye, Jianfeng Liang, Yan Ni, Linhua Tan

**Affiliations:** 1 Department of Surgical ICU, Children’s Hospital, Zhejiang University School of Medicine, National Clinical Research Center for Child Health, Hangzhou, China; 2 Department of Nephrology, Children’s Hospital, Zhejiang University School of Medicine, National Clinical Research Center for Child Health, Hangzhou, China; 3 Department of Medical Statistics, Children’s Hospital, Zhejiang University School of Medicine, National Clinical Research Center for Child Health, Hangzhou, China; 4 Children’s Hospital, Zhejiang University School of Medicine, National Clinical Research Center for Child Health, Hangzhou, Zhejiang China

## Abstract

**Background:**

The close relationship between bile acid (BA) metabolism and sepsis has been investigated in recent years, as knowledge of the role of the gut microbiome and metabolomics in sepsis has grown and become more comprehensive.

**Methods:**

Patients with sepsis who were admitted to the PICU of the Children’s Hospital, Zhejiang University School of Medicine from January 2016 to December 2021 were enrolled in this study. Preoperative non-infectious pediatric patients undergoing elective surgeries in our hospital’s department of surgery were recruited as controls during the same period. Clinical data were collected and analyzed.

**Results:**

702 children were enrolled, comprising 538 sepsis survivors, 164 sepsis fatalities, and 269 non-infected controls. Statistical analysis revealed that total BA (TBA) increased in both the early and severe stages of pediatric sepsis. In the severe stage, TBA (OR = 2.898, 95% CI 1.946–4.315, *p* < 0.05) was identified as a risk factor for sepsis. A clinical model identified TBA (the cut-off value is >17.95 µmol/L) as an independent predictor of sepsis mortality with an AUC of 0.842 (95% CI 0.800–0.883), sensitivity of 54.9%, specificity of 96.6%, and HR = 7.658 (95% CI 5.575–10.520).

**Conclusions:**

The study showed that elevated TBA was associated with a heightened risk of mortality in pediatric sepsis.

**Impact:**

- Many clinical indicators show differences between children with sepsis and the control group, among which the difference in serum total bile acid levels is the most significant.
- During the hospitalization of the patients, the overall bile acid levels in the sepsis death group were higher and exhibited greater fluctuations compared to the survival group, with significant differences.
- Serum total bile acid levels can serve as effective biomarker for predicting the prognosis of children with sepsis.

## Introduction

Sepsis is an organ dysfunction that can be fatal and is brought on by the body’s inability to regulate its response to infection. It contributes significantly to the global morbidity, mortality, disability, and medical burden among children. Global epidemiological data indicates that the incidence of sepsis is greatest during early childhood.^1^ In 2017, there were an estimated 20.3 million cases of sepsis in children under the age of 5, 4.9 million cases of sepsis in children and adolescents aged 5–19 years, and 23.7 million cases of sepsis in adults aged 20 and above worldwide. The case fatality rate for severe sepsis is as high as 50%.^2^ Timely identification, active intervention, and comprehensive management are crucial components in enhancing outcomes for children suffering from sepsis.

For the early detection and management of pediatric sepsis in clinical practice, improved biomarkers are urgently required due to the distinctive characteristics of children, the disease’s complexity and heterogeneity, and the ambiguous definition of childhood sepsis. Biomarkers have the potential to function as diagnostic instruments for abnormalities or diseases, in addition to providing information regarding disease progression, prognosis, and response to intervention strategies. This capability is crucial in the pursuit of precision medicine.^3^ Over the last few decades, more than 250 biomarkers have been identified and evaluated.^4^ Recent research has revealed that bile acids not only play crucial physiological roles in the digestive system but also function as hormones, binding to multiple bile acid receptors and exerting significant effects on metabolism, inflammation, immune balance, tumorigenesis, aging, and various other biological processes. Furthermore, as our understanding of the impact of gut microbiota in sepsis becomes more comprehensive, there has been a gradual exploration of the intricate connection between bile acid metabolism and sepsis.^5^ During sepsis, the release of inflammatory mediators suppresses the expression of genes encoding hepatic transport proteins, leading to disrupted transport and metabolism of bile acids, resulting in hyperbilirubinemia and bile stasis.^6^ It is important to note that in the presence of elevated circulating bile acids, the nuclear FXR/RXR heterodimer is sequestered in the cytoplasm, resulting in the loss of feedback inhibition of bile acid synthesis.^7^ The elevated BA in the body can be used as DAMPs to activate the NLRP inflammasome signaling pathway, thereby accelerating sepsis progression and high mortality rates.^8^ Bile acids may serve as potential biomarkers for sepsis.^5^

Biomarkers with high sensitivity and specificity, such as interleukin-8 (IL-8), C-reactive protein (CRP), procalcitonin (PCT), D2 polymer(D-dimer), presepsin, brain natriuretic peptide, lactate (Lac), activated partial thromboplastin time (APTT), endocan, and monocyte chemotactic protein-1 (mcp-1) each possible pediatric sepsis biomarkers have their own characteristics and application differences. PCT and CRP continue to be the most frequently assessed biomarkers for patients with sepsis. However, when applied to clinical environments without sepsis, they may exhibit elevated values and frequently fail to furnish dependable prognostications for patients. Consequently, the clinical implementation of numerous biomarkers that presently exhibit promise continues to present obstacles and occasionally generate controversy.^9^ Despite the identification of numerous biomarkers that can be utilized to predict the prognosis and early detection of sepsis, current research on these indicators is primarily laboratory-based, with limited attention paid to clinical applications for sepsis patients. In addition, the aforementioned biomarker studies primarily examined adults and were restricted to children afflicted with sepsis.

Based on previous research, it has been shown that clinical indicators in children with sepsis play a significant role in diagnosing and monitoring the disease process. Therefore, this study hypothesizes to explore the expression of biomarkers such as D-dimer, CRP, PCT, and BA in children with sepsis, and assess their impact on the severity of the disease and prognosis. By examining the correlation between changes in these biomarkers and the condition of children with sepsis, it is hoped to provide a more accurate reference basis for clinical diagnosis and treatment.

## Methods

### Study population and design

This is a retrospective case case-control study of 702 children with sepsis. The inclusion criteria were as follows: (1) The patient met the Phoenix sepsis criteria. (2) Age was ≥28 days and ≤18 years old; and (3) Complete clinical data were available. The exclusion criteria included: (1) Presence with basic diseases such as malignant tumors, metabolic diseases, etc; (2) Within the initial 3 months of enrollment, patients who are prescribed antibiotics, probiotics, or any other medication that could impact the metabolism. At the same time, children of the same age and sex who were hospitalized for elective surgery in our hospital were recruited as controls. Children in this group had conditions such as hernia, adenoid hypertrophy, and hemangioma, all of whom had complete clinical data, no recent infection history, and displayed normal growth and development. This study was approved by the Ethics Committee of the Children’s Hospital Affiliated to Zhejiang University School of Medicine on August 26, 2022, under the application titled “Composition and metabolic characteristics of intestinal microbiota in children with sepsis (2022-IRB-193),” and conducted in accordance with the Helsinki Declaration of 1975.

### Clinical data and patients’ characteristics

Data collection: Common clinical information about children, such as age, sex, BMI, clinical manifestations, prognosis, treatment, and laboratory indicators during hospitalization, as well as demographic information including education, diet, geographical location, and related population statistics, were all collected through the hospital’s electronic medical record system. The critical case score was determined according to the international pediatric critical case scoring standard, including the Phoenix sepsis criteria,^10^ PRISM IV and PCIS.^11^

### Statistics

Statistical analyses were performed using the IBM® SPSS Statistics software version 26. The data were assessed for normal distribution using the Shapiro-Wilk test. Measurement data conforming to a normal distribution are expressed as the mean ± standard deviation (x ± s), and an independent sample *t* test was used for group comparisons. Nonnormally distributed metrological data were represented by the median and quartile spacing (M, Q), and group comparisons were conducted using the Mann–Whitney U test. For group comparisons, counting data are represented by example (%) using the chi-square test or Fisher’s exact probability method. A logistic regression model was used for multivariate regression analysis, and Pearson correlation analysis was used for correlation analysis. Variables with statistically significant differences were included in binary logistic regression analysis to select independent risk factors for sepsis death, clinical prediction models for sepsis prognosis were constructed and clinical prediction probabilities were generated. The receiver operating characteristic curve (ROC) was verified, and an Area Under Curve (AUC) > 0.5 was considered to have predictive value. The closer the value was to 1.0, the greater the prediction accuracy. *P* < 0.05 was considered statistically significant and the confidence interval was 95%.

## Result

### Cohort description

**Fig. 1:**
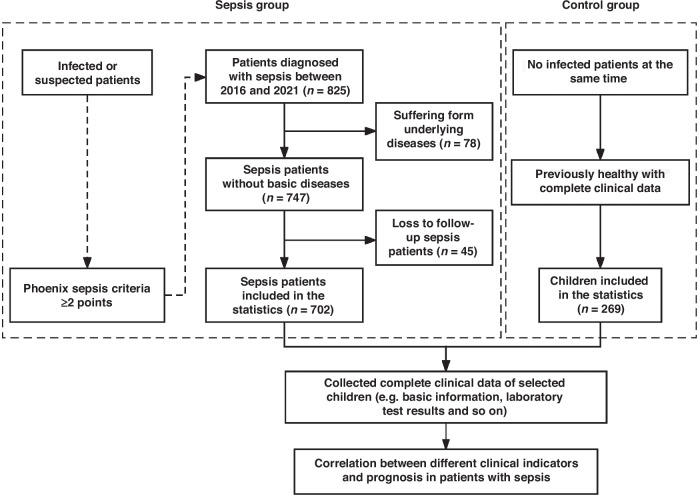
Flowchart of patient selection.

**Fig. 2:**
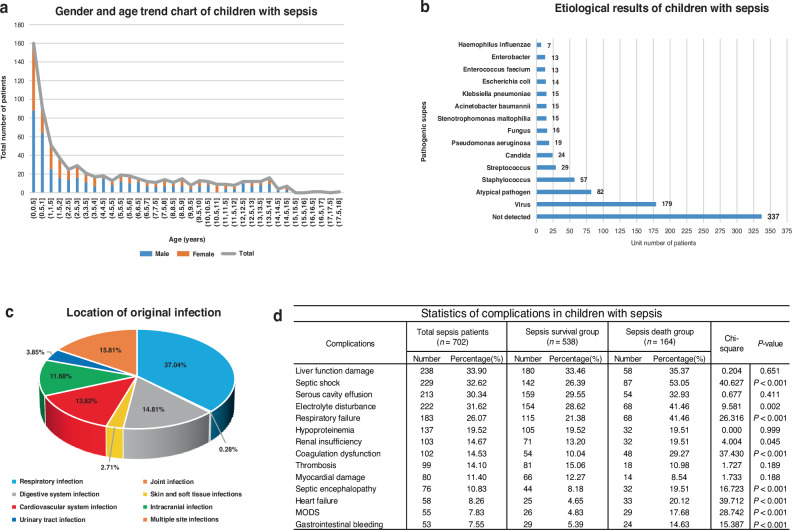
Demographic and clinical basic information of septic children. **a** Age and sex characteristics of children with sepsis. **b** Laboratory etiology results of patients. **c** Infection Site in sepsis. **d** Statistical results of complications of disease subgroups.

A total of 702 children with sepsis and 269 non-infected controls were selected for this study (Fig. 1). Among the children with sepsis, 401 patients (57.12%) were males. The mean age was 4.21 years, with 65.8% of cases occurring in children under 5 years of age (Fig. 2a). The longest length of hospitalization was 168 days, and the median was 14 days. Infection sites in patients with sepsis included 260 respiratory infections (37.04%), 111 multiple-site infections (15.81%), 104 gastrointestinal infections (14.81%), 97 blood-borne infections (13.82%), 82 intracranial infections (11.68%), 27 urinary tract infections (3.85%) (Fig. 2b). Infectious pathogens were isolated in 52% of patients, with the highest detection rate of viruses, while Staphylococcus was the most commonly isolated bacterium, and fungi were mainly Candida (Fig. 2c). In the statistics of complications, septic shock, electrolyte disturbance, respiratory failure, coagulation dysfunction, septic encephalopathy, heart failure, multiple organ dysfunction syndrome (MODS) and gastrointestinal bleeding showed statistically significant differences between the survival and death groups (Fig. 2d). The high incidence season for pediatric sepsis is December, January, and February, with clinical symptoms predominantly presenting as fever (82.76%). Affected children mostly come from the Yangtze River Delta region and receive formal education according to their age. Multiple clinical indicators, including the above-mentioned parameters, as well as non-clinical indicators such as treatment methods, ultrasound and imaging results, and dietary habits, showed no significant statistical differences within the disease subgroups (*P* > 0.05). Therefore, the focus of this study was primarily on laboratory indicators.

According to the prognosis of children with sepsis, all the enrolled children were divided into 3 groups: the sepsis survival group (group 1, 538 patients, 76.64%) and the sepsis death group (group 2, 164 patients, 23.36%). There were 269 children in the non-infected control group (group 3), 144 males (53.53%) and 125 females (46.47%). The mean age was 3.74 years. There was no significant difference in age or sex between the non-infected control group and the sepsis group (*Z* value = −0.472, *χ*^2^ = 1.018, *P* > 0.05). The clinical data of patients and controls were comparable.

### Clinical baseline information was compared between patients in the no-sepsis and sepsis groups

Using the preoperative laboratory indicators of non-infected controls as the baseline level, this research compared the experimental values of patients at different stages of sepsis. We collected all laboratory indicators for children with sepsis throughout their hospitalization, but ultimately selected two groups of indicators for inclusion in the analysis, specifically the laboratory indicators on the day sepsis were diagnosed and laboratory indicators assessed as the most severe stage of disease based on the scoring system and clinical presentation. There were no statistically significant differences observed in age, sex percentage, or body mass index (BMI) between the cases and controls (*P* > 0.05), as indicated in Supplementary Table 1. Abnormal laboratory findings are common in children with sepsis. In the blood gas and electrolyte analysis, pH value, Na^+^ and K^+^ level, and blood glucose (Glu) were significant differences between patients and controls. Hematological parameters including white blood cell count (WBC), Hemoglobin (HB), platelet count (PLT), and CRP in the whole blood and biochemical parameters such as liver function test (albumin (ALB), globulin (GLB), bilirubin (BIL), TBA, Alanine aminotransferase (ALT), aspartate aminotransferase (AST)), kidney function test (serum creatinine (SCR), blood urea nitrogen (BUN), uric acid (UA), Ca^2+^, P^2+^, Mg^2+^) in serum and heart function test (lactate dehydrogenase (LDH)), cardiac marker test (creatine kinase (CK), CK-MB), D-dimer test and inflammatory and immunologic laboratory markers reveal significant disparities. Subsequently, a comparative analysis was conducted between the clinical indicators of the sepsis survival subgroup and the death subgroup. The results indicated that the proportions of age and sex varied between subgroups (Supplementary Table 2). The remainder of the clinical indicators that exhibited significant differences (*P* < 0.05) were included in the logistic regression analysis.

### Multifactorial logistic regression analysis of the risk of death in patients with sepsis

**Fig. 3:**
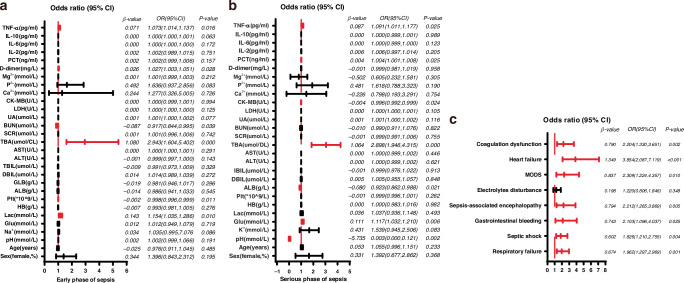
Regression models of disease subgroups, all measures with statistically significant differences are highlighted in red. **a** In the early stages of diagnosis, binary regression analysis was performed on the different indicators obtained from the univariate analysis to select the indicators with statistical differences. **b** Comparison of results of binary regression analysis of laboratory test results detected at the serious stage of pediatric sepsis. **c** Complication binary regression statistics of sepsis survival subgroup and death subgroup.

Clinical indicators of early stage of sepsis, including age, sex percentage, pH, Na^+^, Glu, HB, Lac, PLT, ALB, GLB, direct bilirubin (DBIL), indirect bilirubin (TBIL), TBA, SCR, BUN, UA, LDH, CK-MB, Ca^2+^, P^2+^, Mg^2+^, D-dimer, PCT, IL-2, IL-6, IL-10, and TNF-a levels were selected for multivariate logistic regression (Fig. 3a). The model was evaluated by the Hosmer-Leme show equation, *P* = 0.891, indicated that the clinical prediction model of sepsis prognosis had a good degree of fit. The results showed that PLT (OR = 0.998, 95% CI = 0.996–0.999, *P* = 0.010) and BUN (OR = 0.917,95% CI = 0.844–0.995, *P* = 0.039) were protective factors for the prognosis of children with sepsis, but TBA (OR = 2.943, 95% CI = 1.604–5.402, *P* = 0.000), Lac (OR = 1.154, 95% CI = 1.035–1.286, *P* = 0.010), D-dimer (OR = 1.027, 95% CI = 1.003–1.050, *P* = 0.025), and increased TNF-α (OR = 1.073, 95% CI = 1.014–1.137, *P* = 0.016) were independent prognostic risk factors for children with sepsis. In the serious stage of sepsis, age, sex percentage, pH, K^+^, Glu, Lac, HB, PLT, ALB, DBIL, IBIL, ALT, AST, TBA, SCR, BUN, UA, LDH, Ca^2+^, P^2+^, Mg^2+^, D-dimer, PCT, IL-2, IL-6, IL-10 and TNF-α were selected for multivariate logistic regression (Fig. 3b). The model was evaluated by the Hosmer-Leme show equation, *P* = 0.946. The final results showed that pH (OR = 0.003, 95% CI = 0.000–0.121, *P* = 0.002), ALB (OR = 0.923, 95% CI = 0.862–0.988, *P* = 0.021), and CK-MB (OR = 0.996, 95% CI = 0.992–0.999) were protective factors for the prognosis of sepsis. Glu level (OR = 1.117, 95% CI = 1.032–1.210, *P* = 0.006), TBA (OR = 2.898, 95% CI = 1.946–4.315, *P* = 0.000), PCT (OR = 1.004, 95% CI = 1.001–1.008, *P* = 0.005) and TNF-α (OR = 1.091,95% CI = 1.011, 1.177) were independent prognostic risk factors for pediatric sepsis. In the binary logistic analysis of sepsis complications (Fig. 3c), respiratory failure (OR = 1.962, 95% CI = 1.297–2.969, *P* = 0.001), septic shock (OR = 1.826, 95% CI = 1.210–2.755, *P* = 0.004), gastrointestinal bleeding (OR = 2.103, 95% CI = 1.096–4.037, *P* = 0.025), sepsis-associated encephalopathy (OR = 2.212, 95% CI = 1.265–3.869, *P* = 0.005), MODS (OR = 2.309, 95% CI = 1.224–4.357, *P* = 0.010), heart failure (OR = 3.854, 95% CI = 2.087–7.119, *P* < 0.001) and coagulation dysfunction (OR = 2.204, 95% CI = 1.330–3.651, *P* = 0.002) were predictors of inpatient mortality in critically ill children with sepsis.

### The validation of a predictive model for the risk of death in patients with sepsis

**Fig. 4:**
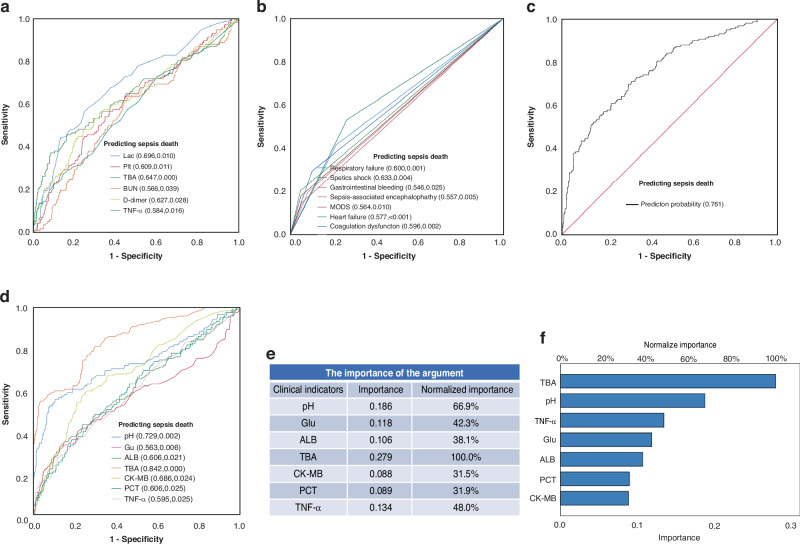
Sepsis prognosis prediction models: ROC analysis and neural network models. **a** The ROC results of statistically different indicators at the early stage of diagnosis of sepsis. **b** The ROC results of statistically different complications of pediatric sepsis. **c** The Joint ROC results of septic shock, Lac, and TBA levels at the early stage of diagnosis of the sepsis. **d** The ROC results of statistically different indicators at the serious stage of the sepsis. **e**, **f** Neural network model of statistically different indicators at the serious stage of the sepsis.

Serum Lac, PLT, TBA, BUN, D-dimer, TNF-α levels of the early stage of sepsis, pH, Glu, ALB, TBA, CK-MB, PCT, and TNF-α levels of the serious stage of sepsis and complications with statistically significant differences were verified by the ROC curve (Fig. 4a–d). The results suggested that lac, TBA level in the early stage of sepsis, and septic shock had maximum AUC in the model, but the final value was still less than 0.7. Given the low value of the single index forecasting model, we set up Lac, TBA and septic shock co-forecasting model, the cut-off value of Lac was 2.75 mmol/L, and the cut-off value of TBA was 13.7umol/L. According to the regression equation P1 = 1/(1 + e(x)), the clinical prediction model equation of sepsis is as follows: P1 = 1/(1 + e(−2.333 + 0.147X^1^ + 0.012X^2^ + 1.068X^3^)), in which X^1^, X^2^, and X^3^ correspond to Lac, TBA, septic shock. The ROC curve was used to verify the prediction model (Fig. 4c), the AUC was 0.761 (95% CI 0.719–0.804) and the cutoff value was 0.204. When P1 > 0.204, the probability of sepsis was greater, the prediction sensitivity was 69.8%, the specificity was 69.1%, the positive predictive value (PPV) was 68.6%, and the negative predictive value (NPV) was 80.8%. On the contrary, the prognosis of sepsis could be accurately predicted by the serum TBA level during the critical stage of the condition; an elevated TBA level was indicative of a greater probability of mortality for the child afflicted with sepsis. According to the ROC curve results (Fig. 4d), the cutoff value of the TBA level was 17.95 µmol/L, the AUC was 0.842 (95% CI 0.800–0.883), the sensitivity was 54.9%, the specificity was 96.6%, and the PPV and NPV of this model were 87.8% and 91.0% respectively. Although TBA is not highly sensitive according to our results, it is highly specific and is a simple laboratory parameter that is more readily available. The neural network model (Fig. 4e, f) shows that the TBA level is the most important among the above indicators, demonstrating its greatest impact on the predictive value of disease prognosis. This further indicates that an increase in bile acid levels will lead to an increase in sepsis mortality. The increase in serum TBA levels can provide a high reference value for the early detection and timely identification of the adverse prognosis of sepsis.

### Clinical significance of serum total bile acid level

#### Clinical utility of a model for risk of death in patients with sepsis

**Fig. 5:**
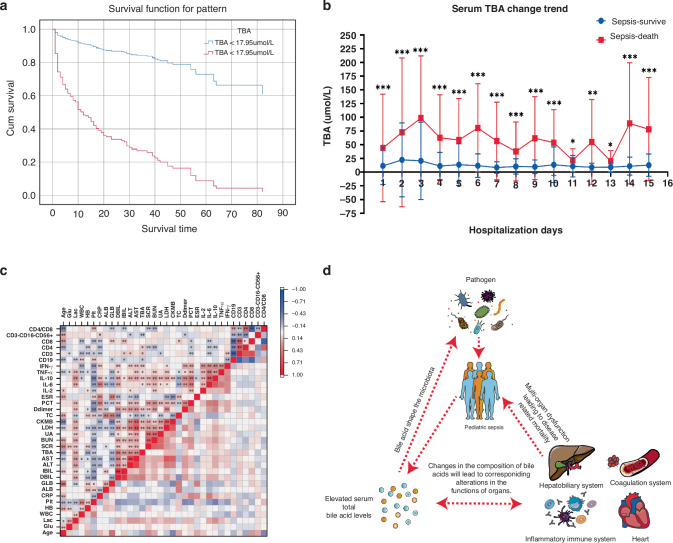
Association between serum bile acid levels and prognosis of sepsis: analysis of clinical indicators and potential mechanistic insights. **a** Cox proportional-hazards model for different bile acid levels. **b** Trend of daily serum total bile acid levels during the stage of sepsis disease, * *P* < 0.05, ** *P* < 0.01, *** *P* < 0.001. **c** The heat map shows the correlation between various clinical indicators in children with sepsis. Red indicates a positive correlation, blue indicates a negative correlation, * *P* < 0.05, ** *P* < 0.01. **d** Sepsis in children caused by pathogenic infections usually leads to an increase in serum total bile acid levels. According to the results shown in (**c**), serum bile acids are closely associated with the inflammatory immune response, coagulation, liver function, and myocardial biomarkers of children with sepsis. Bile acids further impact the prognosis of the disease by interacting with various systems of the host.

The baseline TBA of 702 patients with sepsis was determined in this study. Based on the cutoff value (17.95 µmol/L), every patient was allocated into two distinct groups. As defined by the primary endpoint, the sepsis case fatality rate comprised all-cause mortality that occurred during the patient’s post-admission stay in the PICU, including deaths resulting from treatment discontinuation. According to the Cox risk model assessment (Fig. 5a), it was concluded that TBA level was an independent risk factor for sepsis death (*P* = 0.000). When the serum TBA level ≥ 17.95 µmol/L, the mortality of children with sepsis was significantly increased, HR = 7.658 (95% CI:5.575–10.520), *P* < 0.05.

#### Variation in TBA measurement of sepsis cohort

This study examined the decline and recovery of sepsis survival and mortality-related serum TBA levels during hospitalization. Bile acid levels were significantly higher in children who died from sepsis compared to those who survived the entire hospitalization (Fig. 5b). In contrast, the bile acid concentration of children who survived sepsis exhibited a comparatively stable trend in comparison to the fluctuating bile acid concentration of children who perished from sepsis. Hence, to accurately forecast the prognosis of sepsis in septic children, it is indispensable to dynamically monitor the trend of bile acid change throughout their hospitalization.

#### Correlation of each laboratory indicator in patients with sepsis

Spearman correlation analysis of TBA (Fig. 5c) showed that TBA levels correlated positively with Lac (*r* = 0.208), DBIL (*r* = 0.403), IBIL (*r* = 0.257), ALT (*r* = 0.300), AST (*r* = 0.316), LDH (*r* = 0.146), CK-MB (*r* = 0.174), D-dimer (*r* = 0.114), IL-6 (*r* = 0.083), IL-10 (*r* = 0.107), TNF-α (*r* = 0.084), IFN-γ (*r* = 0.133), CD3 (*r* = 0.113), CD4 (*r* = 0.107), and CD8 (*r* = 0.022). But, it was negatively correlated with HB (*r* = −0.201), PLT (*r* = −0.098), GLB (*r* = −0.144), TC (*r* = −0.140), CD19 (*r* = −0.099), and CD3-CD16 + CD56+ (*r* = −0.128). In conclusion, the level of TBA is closely related to liver function and inflammatory cytokines, which may cause decreased HB and PLT, most commonly seen in severe and critically ill patients, and can cause the progression of the disease.^12^ Based on the Spearman correlation analysis of serum total bile acids and clinical indicators in children with sepsis, we speculate that elevated bile acids in children with sepsis can further impact the prognosis through interactions with various systems within the patient (Fig. 5d).

## Discussion

Sepsis is a global public health issue. Pediatric sepsis often presents with nonspecific symptoms, especially in younger age groups, unlike adult sepsis. Progression to multiple organ failure, shock, and death is rapid. Given that newborns and children account for a significant portion of sepsis-related deaths, early identification and prompt treatment of pediatric sepsis are crucial.^13^ The Phoenix sepsis criteria focus on four organ systems, but other organ dysfunction is vital in pediatric sepsis. The temporal sequence of organ dysfunction and death post-infection doesn’t always indicate causation. Physiological dynamic measurements may better capture patient deterioration than the static or single-point evaluation in the criteria.^10^ Biomarkers are quantifiable indicators that are present during the progression of diseases and the condition of individuals. They aid in the identification of patients, and function as precise and replicable diagnostic agents for treatment.^14^ There’s rising interest in using biomarkers for sepsis diagnosis and monitoring. Most research focuses on diagnostic biomarkers due to treatment guidelines emphasizing early detection. Biomarkers that predict poor outcomes and delineate sepsis subgroups are still needed.^15^

Abnormal liver function is prevalent among ICU patients. Liver function impairment was identified in 33.9% of patients in this study. BA levels exceed the normal range when liver function is impaired, resulting in cytotoxic effects and abnormal elevations in biochemical markers. Consequently, liver function and BA metabolism disorders intertwine in a harmful cycle.^16^ Additionally, bile acids play a crucial role in the body’s immune system. The immune system is vital for tissue repair post-infection, protection against new infections, and clearance of existing infections.^17,18^ In the ileum, active BA absorption sites trigger T-cell-mediated inflammation.^19^ Other bile acids, such as 3-oxoLCA and isoLCA, regulate the immune response by inhibiting TH17 cell function.^20^ Chemokine CXCL16, which is secreted by hepatocytes, exerts regulatory control over the accumulation of NKT cells. This process is notably impacted by the conversion of primary bile acids to secondary BA.^21^ Secondary BA can activate vitamin D receptors, regulate Treg cells in the lamina propria of the colon, and effectively regulate host adaptive immunity.^22^ Prolonged exposure to bile acids in critically ill patients induces immune system damage, inflammation, and subsequent detrimental consequences.^7,23^ The gut barrier is influenced differently by various types of BA, and an imbalance in gut homeostasis can lead to the failure of multiple organs and even death.^24^ Furthermore, BA have been shown in several studies to possess an antimicrobial effect against luminal pathogens.^25^ In our data analysis, there is a certain correlation between BA and cardiac function. Previous studies have also confirmed that BA can affect heart rate and myocardial contractile function. In patients with cirrhosis, heart failure is closely related to the elevated concentration of serum bile acids.^26^ In a mouse model of cholestatic liver injury, elevated levels of BA in hepatocytes are associated with the activation of coagulation and platelets. In children with sepsis, there is also a correlation between BA and platelets as well as D-dimer. These indicators are closely associated with the severity of the disease.^27^

Most studies on BA metabolism in sepsis focus on adults. Elevated plasma BA levels predict adult sepsis mortality, while cholestasis is common in adult sepsis. BAs show promise as prognostic biomarkers for adult sepsis and hold potential for pediatric patients. Research suggests the rare BA TOMCA has diagnostic value and serves as an independent risk factor for neonatal sepsis.^28^ The results of this study indicate that deceased pediatric septic patients have higher levels of TBA, and there is a significant correlation between TBA levels and clinical indicators related to organ function.

### Strengths and limitations

This study offers notable advantages. It is among the few retrospective studies exploring TBA levels in critically ill children with sepsis. Standard counts were used to measure serum TBA levels, with healthy children serving as controls for comparison. BA, as a noninvasive biomarker, stands out for its repeatability and practicality. Nonetheless, certain limitations need to be acknowledged. Prevalence concerns arose due to the limited follow-up evaluations during hospitalization because of the small cohort size, single-center design, and recording only death or discharge outcomes. However, other studies reported similar results on demographics, disease severity, and outcomes.^2,29^ Moreover, only TBA was assessed in this study. However, BA metabolism is highly intricate, with over 60 identified bile acids possibly surpassing 100 in number.^25^ This number likely includes both those that are detrimental to the prognosis of sepsis and those that are advantageous. The overall TBA level can only reflect the approximate level. BA effects are context-dependent and are affected by microbiota, disease state, clinical treatment methods, and diet.^25^ Inconsistent TBA detection baselines among intensive care unit-admitted children may have influenced the findings of this research.

## Conclusion

This study proposes that a TBA level greater than 17.95 µmol/L can be utilized to predict the prognosis of sepsis in children. Survival outcomes, the severity of sepsis, and organ dysfunction are all intricately associated with it. In addition, biomarker kinetics are more useful than single values in predicting sepsis, and continuous measurement of TBA can assist in the comprehension of children’s dynamic conditions. Subsequently, the trend of serum TBA levels in children with sepsis will require larger, more standardized, and unified detection criteria, as well as multicenter, multidisciplinary investigations, and multi-center testing. An additional examination and analysis were conducted on the bile acid composition spectrum of children who had sepsis to investigate the potential correlation between the mechanism of BA metabolism and clinical outcomes in this population.

## Supplementary Information

Supplementary Table 1

Supplementary Table 2

## Data Availability

All data generated or analyzed during this study are included in this published article. The datasets generated during and/or analyzed during the current study are available from the corresponding author upon reasonable request.
